# Bis(μ-nitrito-κ^2^ *O*:*O*)bis­[bis­(1-methyl-1*H*-imidazole-κ*N* ^3^)(nitrito-κ*O*)copper(II)]

**DOI:** 10.1107/S1600536812009804

**Published:** 2012-03-10

**Authors:** Run-Qiang Zhu

**Affiliations:** a Ordered Matter Science Research Center, College of Chemistry and Chemical Engineering, Southeast University, Nanjing 211189, People’s Republic of China

## Abstract

In the binuclear title compound, [Cu_2_(NO_2_)_4_(C_4_H_6_N_2_)_4_], centro­symmetric­ally related complex mol­ecules are linked *via* weak Cu—O inter­actions, forming dimeric units. The Cu^II^ atom displays an elongated square-pyramidal CuN_2_O_3_ coordination geometry with a slight tetra­hedral distortion of the basal plane [maximum deviation = 0.249 (2) Å]. The dihedral angle formed by the imidazole rings is 26.20 (10)°.

## Related literature

The structure of the title compound was determined as part of our ongoing study of potential ferroelectric phase change materials. For general background to ferroelectric compounds with metal-organic framework structures, see: Fu *et al.* (2009 ▶); Ye *et al.* (2006 ▶); Zhang *et al.* (2008 ▶, 2010 ▶). For a related structure, see: Costes *et al.* (1995 ▶). 
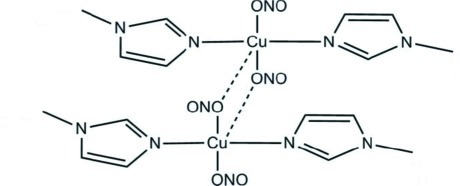


## Experimental

### 

#### Crystal data

[Cu_2_(NO_2_)_4_(C_4_H_6_N_2_)_4_]
*M* *_r_* = 639.55
Triclinic, 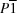

*a* = 7.8281 (16) Å
*b* = 8.4873 (17) Å
*c* = 10.054 (2) Å
α = 80.35 (3)°
β = 77.72 (3)°
γ = 79.46 (3)°
*V* = 635.9 (2) Å^3^
*Z* = 1
Mo *K*α radiation
μ = 1.74 mm^−1^
*T* = 293 K
0.29 × 0.23 × 0.20 mm

#### Data collection

Rigaku SCXmini diffractometer
Absorption correction: multi-scan (*CrystalClear*; Rigaku, 2005 ▶) *T*_min_ = 0.625, *T*_max_ = 0.706
6578 measured reflections
2900 independent reflections
2465 reflections with *I* > 2σ(*I*)
*R*_int_ = 0.035

#### Refinement

*R*[*F* ^2^ > 2σ(*F* ^2^)] = 0.037
*wR*(*F* ^2^) = 0.091
*S* = 1.05
2900 reflections
174 parameters
H-atom parameters constrained
Δρ_max_ = 0.40 e Å^−3^
Δρ_min_ = −0.29 e Å^−3^

### 

Data collection: *CrystalClear* (Rigaku, 2005 ▶); cell refinement: *CrystalClear*; data reduction: *CrystalClear*; program(s) used to solve structure: *SHELXS97* (Sheldrick, 2008 ▶); program(s) used to refine structure: *SHELXL97* (Sheldrick, 2008 ▶); molecular graphics: *DIAMOND* (Brandenburg & Putz, 2005 ▶); software used to prepare material for publication: *SHELXL97*.

## Supplementary Material

Crystal structure: contains datablock(s) I, global. DOI: 10.1107/S1600536812009804/rz2715sup1.cif

Structure factors: contains datablock(s) I. DOI: 10.1107/S1600536812009804/rz2715Isup2.hkl

Additional supplementary materials:  crystallographic information; 3D view; checkCIF report

## Figures and Tables

**Fig. 1.**
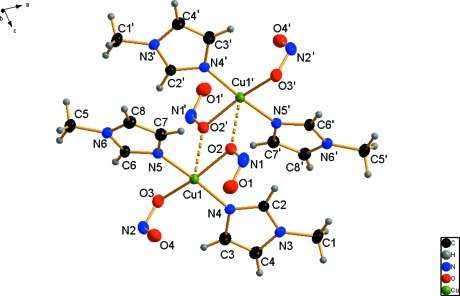
Partial packing diagram of the title compound, with displacement ellipsoids drawn at the 30% probability level. The weak Cu—O interactions are shown as dashed lines. Primed atoms are generated by the symmetry operator: (') 1-x, 1-y, -z.

**Fig. 2.**
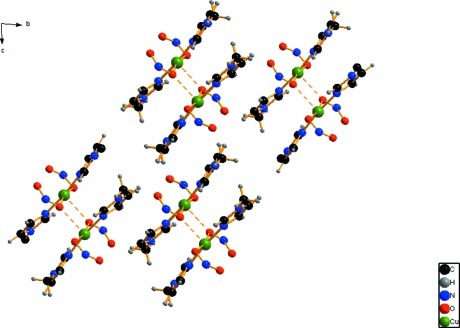
Packing diagram of the title compound. The weak Cu—O interactions are shown as dashed lines.

## supplementary crystallographic information

### Comment

As part of our ongoing study of potential ferroelectric phase change materials we have determined the structures of several copper complexes and examined the changes in their dielectric constants with temperature, which is the usual method for detecting such behaviour (Fu *et al.*, 2009; Ye *et al.*, 2006; Zhang *et al.*, 2008; Zhang *et al.*, 2010). Unfortunately, the dielectric constant of the title compound indicates the onset of a ferroelectric phase change over the range 80–298 K.

As show in Fig. 1, the copper ion adopts an elongated square pyramidal geometry with a slight tetrahedral distortion in the basal plane (maximum deviation 0.249 (2) Å for atom N4) which is primarily associated with the coordination of the nitrite ions (O2—Cu1—O3 = 167.15 (8)°). As observed in a related compound (Costes *et al.*, 1995), this geometry displaces atom O2 from the ideal coordination plane towards the centrosymmetrically related Cu1^i^ copper atom [symmetry code: (i) 1-x, 1-y, -z] resulting in an O2—Cu1 distance of 2.578 (5) Å. While this distance is considerably longer than those in basal plane (Cu1—O2 and Cu1—O3 are 2.0221 (19) and 2.0085 (19) Å, respectively), the direction of the displacement of atom O2 and the orientations of the two nitrite ligands which place both atoms O1 and O3 on the opposite side of the coordination plane, suggest that there is a weak association of one complex molecule with its centrosymmetrically related. The dihedral angle formed by the *trans*-arranged imidazole rings is 26.20 (10)°. The crystal packing (Fig. 2) is governed only by van der Waals interactions.

### Experimental

An aqueous solution of 1-methylimidazole (2.0 g, 25 mmol) and H_2_SO_4_ (12.5 mmol) was treated with CuSO_4_ (250 g, 12.5 mmol). After the mixture was stirred for a few minutes, Ba(NO_2_)_2_ (6.18 g, 25 mmol) was added to give a blue solution. Slow evaporation of the solution following removal of the precipitated BaSO_4_ yielded blue crystals after a few days. M. p. 319–329 K.

### Refinement

All H atoms were placed in geometrically idealized positions and constrained to ride on their parent atoms with C—H = 0.93–0.96 Å, and with *U*_iso_(H) = 1.2 *U*_iso_(C) or 1.5 *U*_iso_(C) for methy H atoms.

### Crystal data

**Table:**

[Cu_2_(NO_2_)_4_(C_4_H_6_N_2_)_4_]	*Z* = 1
*M**_r_* = 639.55	*F*(000) = 326
Triclinic, *P*1	*D*_x_ = 1.670 Mg m^−^^3^
Hall symbol: -P 1	Mo *K*α radiation, λ = 0.71073 Å
*a* = 7.8281 (16) Å	Cell parameters from 2900 reflections
*b* = 8.4873 (17) Å	θ = 2.3–27.5°
*c* = 10.054 (2) Å	µ = 1.74 mm^−^^1^
α = 80.35 (3)°	*T* = 293 K
β = 77.72 (3)°	Prism, blue
γ = 79.46 (3)°	0.29 × 0.23 × 0.20 mm
*V* = 635.9 (2) Å^3^	

### Data collection

**Table:**

Rigaku SCXmini diffractometer	2465 reflections with *I* > 2σ(*I*)
Radiation source: fine-focus sealed tube	*R*_int_ = 0.035
Graphite monochromator	θ_max_ = 27.5°, θ_min_ = 3.0°
ω scans	*h* = −10→10
Absorption correction: multi-scan (*CrystalClear*; Rigaku, 2005)	*k* = −11→11
*T*_min_ = 0.625, *T*_max_ = 0.706	*l* = −13→13
6578 measured reflections	2 standard reflections every 150 reflections
2900 independent reflections	intensity decay: none

### Refinement

**Table:**

Refinement on *F*^2^	Primary atom site location: structure-invariant direct methods
Least-squares matrix: full	Secondary atom site location: difference Fourier map
*R*[*F*^2^ > 2σ(*F*^2^)] = 0.037	Hydrogen site location: inferred from neighbouring sites
*wR*(*F*^2^) = 0.091	H-atom parameters constrained
*S* = 1.05	*w* = 1/[σ^2^(*F*_o_^2^) + (0.0423*P*)^2^ + 0.1143*P*] where *P* = (*F*_o_^2^ + 2*F*_c_^2^)/3
2900 reflections	(Δ/σ)_max_ = 0.001
174 parameters	Δρ_max_ = 0.40 e Å^−^^3^
0 restraints	Δρ_min_ = −0.29 e Å^−^^3^

### Special details

**Table:**

Geometry. All e.s.d.'s (except the e.s.d. in the dihedral angle between two l.s. planes) are estimated using the full covariance matrix. The cell e.s.d.'s are taken into account individually in the estimation of e.s.d.'s in distances, angles and torsion angles; correlations between e.s.d.'s in cell parameters are only used when they are defined by crystal symmetry. An approximate (isotropic) treatment of cell e.s.d.'s is used for estimating e.s.d.'s involving l.s. planes.
Refinement. Refinement of *F*^2^ against ALL reflections. The weighted *R*-factor *wR* and goodness of fit *S* are based on *F*^2^, conventional *R*-factors *R* are based on *F*, with *F* set to zero for negative *F*^2^. The threshold expression of *F*^2^ > σ(*F*^2^) is used only for calculating *R*-factors(gt) *etc*. and is not relevant to the choice of reflections for refinement. *R*-factors based on *F*^2^ are statistically about twice as large as those based on *F*, and *R*- factors based on ALL data will be even larger.

### Fractional atomic coordinates and isotropic or equivalent isotropic displacement parameters (Å^2^)

**Table:**

	*x*	*y*	*z*	*U*_iso_*/*U*_eq_	
C1	0.7413 (4)	0.2449 (4)	0.4825 (3)	0.0549 (8)	
H1A	0.7735	0.1313	0.4761	0.082*	
H1B	0.7220	0.2619	0.5769	0.082*	
H1C	0.8349	0.3012	0.4302	0.082*	
C2	0.5675 (3)	0.4011 (3)	0.3090 (3)	0.0365 (6)	
H2	0.6629	0.4389	0.2480	0.044*	
C3	0.3061 (4)	0.3554 (4)	0.4028 (3)	0.0496 (8)	
H3	0.1847	0.3553	0.4178	0.059*	
C4	0.4134 (4)	0.2788 (4)	0.4889 (3)	0.0520 (8)	
H4	0.3803	0.2185	0.5738	0.062*	
C5	−0.1244 (4)	0.9207 (4)	−0.2032 (3)	0.0490 (8)	
H5A	−0.2148	0.8597	−0.1523	0.074*	
H5B	−0.0952	0.8978	−0.2964	0.074*	
H5C	−0.1667	1.0341	−0.2020	0.074*	
C6	0.0619 (3)	0.7480 (3)	−0.0451 (3)	0.0341 (6)	
H6	−0.0165	0.6746	−0.0090	0.041*	
C7	0.2892 (4)	0.8716 (3)	−0.0865 (3)	0.0384 (6)	
H7	0.3984	0.8980	−0.0837	0.046*	
C8	0.1763 (4)	0.9545 (3)	−0.1664 (3)	0.0411 (6)	
H8	0.1925	1.0480	−0.2277	0.049*	
N1	0.6145 (3)	0.7315 (3)	0.0924 (3)	0.0485 (6)	
N3	0.5793 (3)	0.3061 (3)	0.4283 (2)	0.0376 (5)	
N2	−0.0346 (3)	0.5775 (3)	0.2749 (3)	0.0495 (7)	
N4	0.4024 (3)	0.4338 (3)	0.2893 (2)	0.0334 (5)	
N5	0.2167 (3)	0.7405 (2)	−0.0090 (2)	0.0314 (5)	
N6	0.0336 (3)	0.8755 (3)	−0.1406 (2)	0.0355 (5)	
O1	0.5052 (3)	0.7955 (3)	0.1808 (2)	0.0541 (5)	
O2	0.5618 (2)	0.6202 (2)	0.04605 (19)	0.0402 (4)	
O3	0.0807 (3)	0.4952 (2)	0.1912 (2)	0.0422 (5)	
O4	0.0182 (3)	0.6897 (3)	0.3082 (2)	0.0542 (6)	
Cu1	0.31394 (4)	0.57713 (4)	0.13253 (3)	0.02903 (12)	

### Atomic displacement parameters (Å^2^)

**Table:**

	*U*^11^	*U*^22^	*U*^33^	*U*^12^	*U*^13^	*U*^23^
C1	0.0475 (18)	0.067 (2)	0.0476 (18)	0.0037 (15)	−0.0230 (15)	0.0039 (16)
C2	0.0324 (13)	0.0429 (16)	0.0316 (14)	−0.0020 (11)	−0.0056 (11)	−0.0021 (11)
C3	0.0389 (15)	0.063 (2)	0.0424 (17)	−0.0159 (14)	−0.0080 (13)	0.0158 (14)
C4	0.0492 (18)	0.066 (2)	0.0363 (16)	−0.0152 (15)	−0.0087 (14)	0.0143 (14)
C5	0.0434 (16)	0.0549 (19)	0.0458 (17)	0.0069 (14)	−0.0183 (14)	−0.0016 (14)
C6	0.0320 (13)	0.0338 (14)	0.0361 (14)	−0.0022 (10)	−0.0103 (11)	−0.0019 (11)
C7	0.0386 (14)	0.0354 (15)	0.0410 (15)	−0.0103 (11)	−0.0087 (12)	0.0016 (12)
C8	0.0473 (16)	0.0341 (15)	0.0391 (15)	−0.0068 (12)	−0.0096 (13)	0.0051 (12)
N1	0.0425 (14)	0.0497 (16)	0.0550 (16)	−0.0166 (12)	−0.0174 (12)	0.0094 (13)
N3	0.0383 (12)	0.0435 (13)	0.0294 (11)	−0.0013 (10)	−0.0103 (10)	−0.0004 (9)
N2	0.0326 (13)	0.0622 (18)	0.0475 (15)	−0.0074 (12)	−0.0065 (11)	0.0092 (13)
N4	0.0328 (11)	0.0378 (12)	0.0275 (11)	−0.0043 (9)	−0.0053 (9)	−0.0007 (9)
N5	0.0299 (11)	0.0318 (11)	0.0313 (11)	−0.0036 (9)	−0.0056 (9)	−0.0023 (9)
N6	0.0368 (12)	0.0352 (12)	0.0324 (11)	0.0020 (9)	−0.0093 (9)	−0.0032 (9)
O1	0.0652 (14)	0.0489 (13)	0.0529 (13)	−0.0133 (11)	−0.0137 (11)	−0.0116 (10)
O2	0.0361 (10)	0.0439 (11)	0.0383 (10)	−0.0065 (8)	−0.0062 (8)	0.0008 (8)
O3	0.0395 (10)	0.0432 (11)	0.0450 (11)	−0.0135 (9)	−0.0125 (9)	0.0041 (9)
O4	0.0547 (13)	0.0539 (14)	0.0501 (13)	−0.0016 (11)	−0.0062 (10)	−0.0076 (11)
Cu1	0.02605 (17)	0.03196 (19)	0.02820 (18)	−0.00537 (12)	−0.00609 (12)	0.00075 (12)

### Geometric parameters (Å, º)

**Table:**

C1—N3	1.460 (4)	C6—N5	1.325 (3)
C1—H1A	0.9600	C6—N6	1.341 (3)
C1—H1B	0.9600	C6—H6	0.9300
C1—H1C	0.9600	C7—C8	1.344 (4)
C2—N4	1.321 (3)	C7—N5	1.385 (3)
C2—N3	1.339 (3)	C7—H7	0.9300
C2—H2	0.9300	C8—N6	1.363 (3)
C3—C4	1.341 (4)	C8—H8	0.9300
C3—N4	1.370 (3)	N1—O1	1.219 (3)
C3—H3	0.9300	N1—O2	1.286 (3)
C4—N3	1.356 (4)	N2—O4	1.225 (3)
C4—H4	0.9300	N2—O3	1.286 (3)
C5—N6	1.466 (3)	N4—Cu1	1.989 (2)
C5—H5A	0.9600	N5—Cu1	1.985 (2)
C5—H5B	0.9600	O2—Cu1	2.0221 (19)
C5—H5C	0.9600	O3—Cu1	2.0085 (19)
			
N3—C1—H1A	109.5	N5—C7—H7	125.5
N3—C1—H1B	109.5	C7—C8—N6	107.0 (2)
H1A—C1—H1B	109.5	C7—C8—H8	126.5
N3—C1—H1C	109.5	N6—C8—H8	126.5
H1A—C1—H1C	109.5	O1—N1—O2	114.3 (2)
H1B—C1—H1C	109.5	C2—N3—C4	107.2 (2)
N4—C2—N3	111.1 (2)	C2—N3—C1	126.1 (2)
N4—C2—H2	124.4	C4—N3—C1	126.8 (2)
N3—C2—H2	124.4	O4—N2—O3	114.5 (2)
C4—C3—N4	109.7 (3)	C2—N4—C3	105.2 (2)
C4—C3—H3	125.2	C2—N4—Cu1	126.73 (18)
N4—C3—H3	125.2	C3—N4—Cu1	127.94 (19)
C3—C4—N3	106.8 (2)	C6—N5—C7	105.6 (2)
C3—C4—H4	126.6	C6—N5—Cu1	125.75 (18)
N3—C4—H4	126.6	C7—N5—Cu1	128.63 (18)
N6—C5—H5A	109.5	C6—N6—C8	107.5 (2)
N6—C5—H5B	109.5	C6—N6—C5	125.5 (2)
H5A—C5—H5B	109.5	C8—N6—C5	127.0 (2)
N6—C5—H5C	109.5	N1—O2—Cu1	115.28 (17)
H5A—C5—H5C	109.5	N2—O3—Cu1	114.51 (17)
H5B—C5—H5C	109.5	N5—Cu1—N4	173.10 (8)
N5—C6—N6	110.9 (2)	N5—Cu1—O3	90.30 (8)
N5—C6—H6	124.6	N4—Cu1—O3	89.99 (9)
N6—C6—H6	124.6	N5—Cu1—O2	90.40 (8)
C8—C7—N5	109.1 (2)	N4—Cu1—O2	90.85 (8)
C8—C7—H7	125.5	O3—Cu1—O2	167.15 (8)
			
N4—C3—C4—N3	−1.2 (4)	C7—C8—N6—C5	179.9 (3)
N5—C7—C8—N6	−0.5 (3)	O1—N1—O2—Cu1	−0.8 (3)
N4—C2—N3—C4	−0.9 (3)	O4—N2—O3—Cu1	−1.9 (3)
N4—C2—N3—C1	179.4 (3)	C6—N5—Cu1—O3	−9.1 (2)
C3—C4—N3—C2	1.3 (3)	C7—N5—Cu1—O3	168.4 (2)
C3—C4—N3—C1	−179.0 (3)	C6—N5—Cu1—O2	158.1 (2)
N3—C2—N4—C3	0.2 (3)	C7—N5—Cu1—O2	−24.4 (2)
N3—C2—N4—Cu1	177.04 (17)	C2—N4—Cu1—O3	168.7 (2)
C4—C3—N4—C2	0.7 (4)	C3—N4—Cu1—O3	−15.1 (3)
C4—C3—N4—Cu1	−176.2 (2)	C2—N4—Cu1—O2	1.6 (2)
N6—C6—N5—C7	0.1 (3)	C3—N4—Cu1—O2	177.7 (2)
N6—C6—N5—Cu1	178.14 (16)	N2—O3—Cu1—N5	−82.48 (18)
C8—C7—N5—C6	0.3 (3)	N2—O3—Cu1—N4	90.62 (18)
C8—C7—N5—Cu1	−177.67 (19)	N2—O3—Cu1—O2	−175.6 (3)
N5—C6—N6—C8	−0.5 (3)	N1—O2—Cu1—N5	90.08 (18)
N5—C6—N6—C5	−179.8 (2)	N1—O2—Cu1—N4	−83.13 (18)
C7—C8—N6—C6	0.6 (3)	N1—O2—Cu1—O3	−176.8 (3)

## References

[bb1] Brandenburg, K. & Putz, H. (2005). *DIAMOND* Crystal Impact GbR, Bonn, Germany.

[bb2] Costes, J. P., Dahan, F., Ruiz, J. & Laurent, J. P. (1995). *Inorg. Chim. Acta*, **239**, 53–59.

[bb3] Fu, D.-W., Ge, J.-Z., Dai, J., Ye, H.-Y. & Qu, Z.-R. (2009). *Inorg. Chem. Commun.* **12**, 994–997.

[bb4] Rigaku (2005). *CrystalClear* Rigaku Corporation, Tokyo, Japan.

[bb5] Sheldrick, G. M. (2008). *Acta Cryst.* A**64**, 112–122. 10.1107/S0108767307043930 18156677

[bb6] Ye, Q., Song, Y.-M., Wang, G.-X., Chen, K. & Fu, D.-W. (2006). *J. Am. Chem. Soc.* **128**, 6554–6555. 10.1021/ja060856p 16704244

[bb7] Zhang, W., Xiong, R.-G. & Huang, S.-P. D. (2008). *J. Am. Chem. Soc.* **130**, 10468–10469. 10.1021/ja803021v 18636707

[bb8] Zhang, W., Ye, H.-Y., Cai, H.-L., Ge, J.-Z. & Xiong, R.-G. (2010). *J. Am. Chem. Soc.* **132**, 7300–7302. 10.1021/ja102573h 20459097

